# Variation in size and shape of toxin glands among cane toads from native-range and invasive populations

**DOI:** 10.1038/s41598-020-80191-7

**Published:** 2021-01-13

**Authors:** Cameron M. Hudson, Gregory P. Brown, Ryann A. Blennerhassett, Richard Shine

**Affiliations:** 1 Department of Biological Sciences, Macquarie University, Sydney, NSW 2109 Australia; 2 Department of Fish Ecology and Evolution, Eawag, Swiss Federal Institute of Aquatic Science and Technology, Center of Ecology, Evolution and Biochemistry, Seestrasse 79, 6047 Kastanienbaum, Switzerland; 3 School of Life and Environmental Sciences, University of Sydney, Sydney, NSW 2006 Australia; 4 Rollins College, Winter Park, FL 32789 USA

**Keywords:** Ecology, Evolution

## Abstract

If optimal investment in anti-predator defences depends on predation risk, invading new regions (and thus, encountering different predators) may favour shifts in that investment. Cane toads offer an ideal system to test this prediction: expensive anti-predator toxins are stored mainly in parotoid glands whose dimensions are easy to measure, and toad invasions have changed the suites of predators they encounter. Although plasticity may influence parotoid morphology, comparisons between parents and progeny revealed that gland dimensions were highly heritable. That heritability supports the plausibility of an evolved basis to variation in gland dimensions. Measurements of 3779 adult toads show that females have larger glands than males, invasive populations have larger glands than in the native-range, and that parotoid sexual size dimorphism varies strongly among invaded areas. Geographic variation in parotoid morphology may be driven by predation risk to both adult toads and offspring (provisioned with toxins by their mother), with toxins allocated to eggs exacerbating the risk of cannibalism but reducing the risk of interspecific predation. Investment into chemical defences has evolved rapidly during the cane toad’s international diaspora, consistent with the hypothesis that organisms flexibly adjust resource allocation to anti-predator tactics in response to novel challenges.

## Introduction

Vertebrates exhibit a variety of anti-predator adaptations^1–3^, but the costs associated with anti-predator phenotypes (e.g. synthesis of chemical defences, weaponry, behaviour) mean that we expect organisms to invest in those defences only if they provide a net benefit to fitness^4–6^. This cost–benefit trade-off is affected by local environmental conditions, such that defensive investments are worthwhile if vulnerability to predation strongly affects an individual’s survival and/or breeding success, and if that vulnerability can be decreased by greater investment in anti-predator adaptations^2,6,7^. If these conditions do not apply, fitness can be enhanced by allocating fewer resources to costly defences^8^ and as a result, anti-predator phenotypes may experience reduced individual fitness when predators are absent^4,9^. Thus, models predict dynamic investment into anti-predator defences in response to environmentally variable factors (such as the effectiveness of defences, and the influence of predation risk on fitness^7,10^). Additionally, investment into anti-predator defences may trade off with mobility, such that a fitness benefit to rapid dispersal may enforce a reduction in weaponry, or favour a shift in the packaging of anti-predator substances in ways that reduce negative impacts on dispersal^11^. To test these ideas, we need a species for which investment into anti-predator defence is expensive; that is exposed to a range of predator threats that differ geographically; that differs geographically in rates of dispersal; and in which it is easy to quantify investment in anti-predator defences.

**Figure 1:**
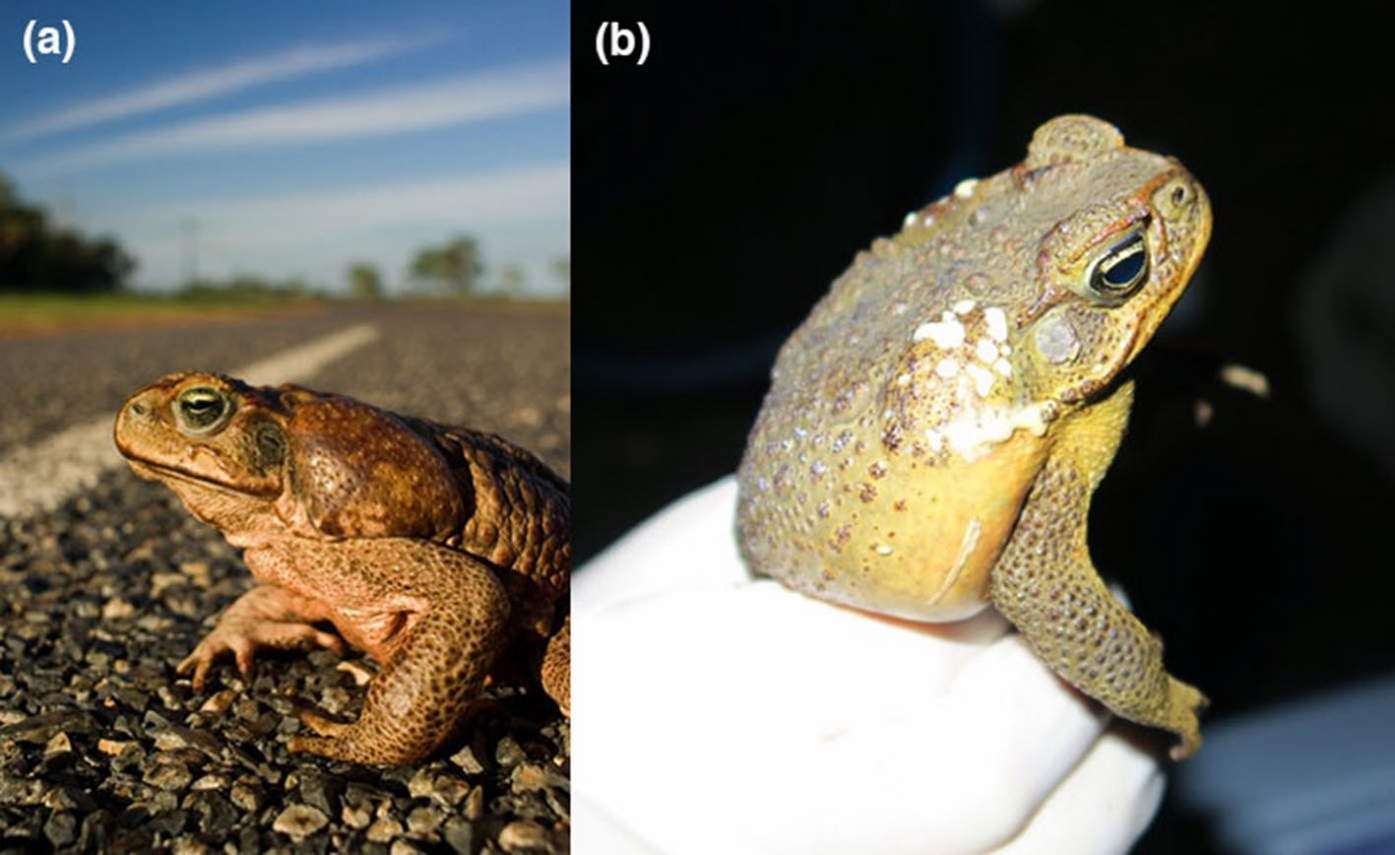
Parotoid macroglands in adult cane toads. **(a)** An adult female male cane toad showing parotoid gland, and **(b)** an adult male cane toad exuding toxin from parotoid glands following capture. Photographs by Cameron Hudson.

Cane toads (*Rhinella marina*) provide an excellent study system in these respects. Like many amphibians, toads synthesise potent chemical defences in the form of steroidal bufotoxins^12–14^ that are energetically expensive to produce^15,16^. Quantifying investment into those chemicals is facilitated by the fact that most toad toxins are produced and stored in parotoid (shoulder) macroglands, whose dimensions can be easily and accurately measured on live animals^17,18^ (Fig. 1). Importantly, cane toads have invaded many areas outside their native range, bringing them into contact with predators that have not co-evolved with bufonids and hence, lack the physiological capacity to tolerate the distinctive toxins produced by these anurans^19,20^. That lack of co-evolutionary history may affect optimal levels of investment into chemical defences. For example, a toxic toad in its native range is likely to be confronted with predators that either are unaffected by its toxic arsenal (small genetic changes to predator physiology confer many thousandfold resistance^20^) or have evolved to exclude large toads from the diet^21,22^. Thus, increased investment into toxin production may confer little benefit to toad fitness in the native range. In contrast, greater investment into chemical defences may enhance survival in the invaded range, because toad-naïve predators cannot tolerate bufonid toxins^19^. In keeping with that hypothesis, interspecific comparisons within the Bufonidae show that possession of potent chemical defences is a significant correlate of range expansion^23^.

In the case of the cane toad, the invaded range in Australia contains many predators that are susceptible to the toxin, and willing to attack adult toads (especially at the invasion front^19^), favouring increased allocation of resources to toxin production. Additionally, cane toads in many parts of Australia are highly dispersive, stimulating rapid evolution of morphological traits (such as relative limb lengths) associated with higher mobility^24^. In contrast, cane toads in Hawai’i are relatively sedentary^25^ and the colonized area contains few species that are predators of adult toads^26^, favouring reduced investment. However, toxins do not protect only adult (terrestrial-phase) toads; reproducing female toads allocate toxins to eggs to protect aquatic stages also^27,28^. As a result, the magnitude of toxin stores in reproductive female toads may comprise two components: that needed for defence of the adult toad, and that needed to provision her offspring.

Toads can change the magnitude of their investment into defensive chemicals via two mechanisms: adaptation and phenotypic plasticity. Under the former mechanism, the size of parotoid glands is expected to show significant heritability; gland sizes vary geographically because individuals with specific sizes of glands survive, and pass that trait on to their progeny. The alternative mechanism, phenotypic plasticity, requires an individual toad to modify its investment into toxin production based on circumstances that it encounters earlier in life. For example, a larval cane toad that is exposed to cues predicting high predation risk develops larger parotoid glands after metamorphosis^29^. Both canalised adaptation and plasticity can increase individual fitness, and can co-occur. Phenotypic plasticity in toxin investment is best demonstrated with experimental studies^30–33^, whereas the likelihood of longer-term selection on parotoid morphology can be clarified by quantifying heritability of this trait.

To test these ideas, we measured the sizes of parotoid glands in field-caught cane toads from the species’ native range in South America, as well as invasive populations on three Hawai’ian Islands and in four regions of Australia. Because climates diverge between windward and leeward shores within each island, and the two sides are separated by inhospitable habitat (making it more difficult for toads to move from one side of an island to another), we treated the “wet” (windward) and “dry” (leeward) sides of each Hawai'ian island as a separate sampling unit. We also bred and raised toads from three Australian populations under standard (“common garden”) conditions, to measure heritability of parotoid morphology.

## Results

The relationship between ln parotoid area and ln SVL was linear, with SVL explaining 75.4% of the variance in ln parotoid area (t = 106.52, 1 df, P < 0.0001). Our measure of parotoid shape (width divided by length) was significantly (P < 0.0001) correlated with our measure of overall relative parotoid size (residual scores from the linear regression of ln parotoid area to ln SVL) but explained very little variance in the latter trait (r^2^ = 0.006). Thus, we treat relative parotoid size and parotoid shape as separate dependent variables in the analyses below.

### Variation in morphology of the parotoid macroglands among field-collected adult toads

**Figure 2:**
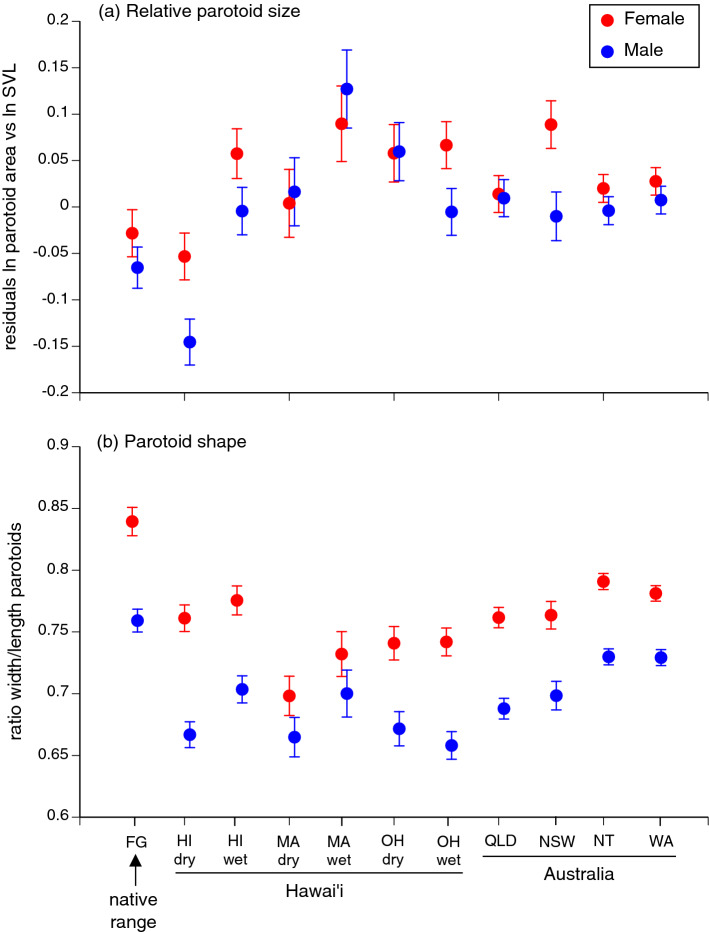
Geographic variation in the size **(a)** and shape **(b)** of parotoid macroglands in field-collected cane toads (*Rhinella marina*). *FG* French Guiana, *HI* Hawai’i, *MA* Maui, *OH* O’ahu, *QLD* Queensland, *NSW* New South Wales, *NT* Northern Territory, *WA* Western Australia. Statistical tests to calculate means derived using JMP 14 software (SAS Institute, Cary, NC).

In seven of the 11 regions that we sampled, female toads had larger parotoid glands than did males at the same body length. At three other sites dimorphism was minimal, and at one site (Maui wet-side) the dimorphism was reversed (larger parotoids in males than females: Fig. 2a). The glands of females were rounder in shape (greater width relative to length) than were those of males, at all sites (Fig. 2b). However, the magnitude of sexual dimorphism in parotoid morphology differed significantly among populations (interaction region*sex from ANOVA, for size F_10,3669_ = 4.70, P < 0.001; for shape F_10,3681_ = 3.06, P < 0.001). Thus, we examined geographic patterns separately in the two sexes, as well as in the combined dataset.

#### Parotoid size

Relative to body length, toads from the native range had smaller parotoids than did conspecifics from any of the invasive populations except those on the dry side of the large island of Hawai’i (Fig. 2a). Parotoids were much larger in other sites within Hawai’i (especially in samples from the wet regions) and in the coolest-climate population in Australia (NSW: see Fig. 2a). Sexual dimorphism in parotoid size was low in the native range (2.3% difference in scores), high in Hawai’i (dry-side 9.2% divergence, wet-side 6.1%) and NSW (9.2%), variable in O’ahu (dry-side < 0.1%, wet-side 7.2%) and low in Maui (dry-side 2.0%, wet-side 3.7%) and in all tropical populations within Australia (0.2 to 2.2% in QLD, NT, WA: Fig. 2a). Because of the significant interaction between sex and region (above), we examined data separately by sex. For females, geographic variation in relative size of the parotoids was significant (F_10,37.63_ = 2.29, P < 0.04), but with no significant posthoc Tukey tests (all comparisons P > 0.05). In males, relative parotoid size also differed significantly among regions (F_10,39.7_ = 6.98, P < 0.0001; posthoc tests show that wet-side Maui and dry-side O’ahu toads differed significantly from Hawai’i dry-side conspecifics).

#### Parotoid shape

Broadly, sexual dimorphism in shape of the parotoids was low in the native range (6% difference in scores), variable in Hawai’i (3.3 to 8.3%), high in Queensland (7.4%), and decreased over the course of the Australian invasion, such that invasion-front populations exhibited similar sexual dimorphism in this trait as did native-range toads (e.g., 5.9% in NT, 5.2% in WA toads: Fig. 2b). Looking only at female toads (because of the significant sex*region interaction, see above), we found significant geographic variation in shape of the parotoids (F_10,41.24_ = 5.53, P < 0.0001; posthoc tests show that parotoids were wider relative to length in toads from French Guiana than in most other groups (QLD, Hawai’i dry-side, O’ahu and Maui both sides), and Maui dry-side toads had significantly smaller parotoids than did those from the native range, NT, WA, NSW or Hawai’ian wet-side conspecifics. Shape variation was also significant in male toads (F_10,44.58_ = 9.83, P < 0.0001), with parotoids significantly more rounded in French Guianan animals than in those from QLD, both sides of O’ahu and dry sides of Hawai’i and O’ahu. Toads from the latter three areas also had significantly smaller parotoids than did toads from WA and the NT.

Across both sexes and in both wild-caught and captive-raised toads, parotoid glands were more rounded in individuals whose limbs were relatively short compared to the body, based on linear regression comparing our parotoid shape index to residual scores from the general linear regression of ln limb length vs. ln SVL. Although limb length explained relatively little variation in parotoid shape in some groups, the relationship was statistically significant for all comparisons (wild-caught females arm length N = 1804, r^2^ = 0.01, P < 0.0001, leg length N = 1907, r^2^ = 0.04, P < 0.0001; wild-caught males arm length N = 1907, r^2^ = 0.04, P < 0.0001, leg length N = 1907, r^2^ = 0.05, P < 0.0001; captive-raised females arm length N = 66, r^2^ = 0.20, P < 0.0002, leg length N = 65, r^2^ = 0.29, P < 0.0001; captive-raised males arm length N = 86, r^2^ = 0.12, P < 0.0015, leg length N = 86, r^2^ = 0.10, P < 0.0025).

### Effect of toxin expulsion on dimensions of the parotoids

ANCOVA (with SVL as covariate to remove body size effects) indicated that when measured five days post-manipulation, parotoid size and shape were indistinguishable between toads from which we had manually expelled toxin from the glands compared to sham-manipulated controls (both F_1,32_ < 1.42, both P > 0.24).

### Assessment of heritability of parotoid morphology

Data from 61 parents and 317 offspring yielded heritability estimates of 0.32 (SE = 0.09) for relative parotoid size and 0.26 (SE = 0.09) for parotoid shape. Repeatability estimates for relative parotoid size and parotoid shape (i.e., ontogenetic consistency of individuals measured at multiple ages) were 0.57 (SE = 0.04) and 0.49 (SE = 0.04), respectively.

## Discussion

Cane toads provide an excellent system in which to study the factors influencing investment into anti-predator defences: the toad’s toxins are expensive to produce^16^, the magnitude of toxin stores is easily quantified^17^, and recent range expansions have modified the toad’s rate of dispersal as well as its exposure to predation in both aquatic and terrestrial phases of the life-history^19^. Variation in dimensions of the parotoid macroglands among individuals is not attributable to recent expulsion of toxins (i.e., as measured over 5 days) and exhibits significant heritability (present study), consistent with the hypothesis that geographic variation in gland morphology reflects adaptive responses to novel challenges. Our sampling design does not allow us to determine whether the heritability of parotoid dimensions is underpinned by genetic versus epigenetic changes.

The most notable changes in shape of the parotoid glands are the evolution of a more elongated parotoid in invasive populations (relative to the situation in the native range) and a decrease in the degree of sexual dimorphism in shape as toads colonized the Australian tropics (Fig. 2b). The former shift might be due either to founder effects (genetic drift) or to novel selective forces. The latter shift mirrors evolutionary changes in relative head size (and the decline in sexual dimorphism in that trait) that has taken place in Australia, perhaps as an adaptation to increased rates of dispersal^34^. Consistent with that hypothesis, length of the limbs relative to the body (a trait that affects dispersal speed, and has evolved rapidly in invasive populations of cane toads^35–37^) was linked to parotoid shape. A large rounded parotoid gland extends well down behind the shoulder, and thus may interfere with mobility of the forelimbs (see Fig. 1a). Under this interpretation, the evolution of greater mobility in invasive populations of cane toads was accompanied by elongation of the parotoid glands as part of a suite of morphological features related to enhanced mobility (see^36,37^ for other traits). Such a shift might have evolved either through adaptation (a fitness benefit to higher rates of dispersal^38^) or through spatial sorting (accumulation of dispersal-enhancing genes at an expanding range edge^39^).

Geographic variation in relative size of the parotoids exhibits more complex patterns (Fig. 2a). Parotoids were larger in the invaded range than in the native range, and varied in size even among invaded sites within both Hawai’i and Australia. These results support Phillips and Shine’s^40^ demonstration of geographic variation in parotoid size within Australian toads, but do not reveal a strong cline associated with invasion history (*contra* the previous analysis).

Variation in parotoid size may be driven at least partly by phenotypic plasticity; exposure to predation cues during larval life stimulated recently metamorphosed cane toads in Australia to develop larger parotoid glands^29^. However, subsequent work in which we have raised toads to maturity revealed no significant difference in parotoid sizes of adult toads as a function of exposure to predation cues during larval life (Sharma et al., unpublished data), suggesting that impacts of larval experience on parotoid dimensions do not persist through to adulthood. High repeatability of parotoid measures in our study of captive-raised toads indicate that the morphology (size and shape) of the glands were consistent across an individual’s lifetime. Additionally, we found significant heritability of parotoid sizes and shapes among offspring raised in standardised conditions (above), a result that would not be expected if gland morphology was sensitive to disruption by environmental factors (although such effects likely were minimised in our study, because offspring were raised under standardised conditions). Our estimates of heritability (26 to 32%) are higher than those we have calculated for other morphological traits of these toads^37^, but are similar to published estimates of heritability of morphological traits more generally^41^.

Even if variation in parotoid size is driven by heritable factors, it might not be adaptive. Biological invasions often involve successive founder effects, increasing the likelihood that gene frequencies will be affected by non-adaptive processes such as genetic drift^42^. In keeping with that scenario, the Hawai'ian populations were founded by a maximum of 150 individuals, and the Australian populations by a maximum of 101 animals^43^. However, existing populations both in Hawai’i and Australia contain thousands or millions of individuals, reducing the impact of random effects on gene frequencies. Thus, the consistent pattern for smaller and more rounded parotoids in the native range than in the invaded range (see Fig. 2a) suggests that geographic variation in parotoid size likely is driven at least partly by deterministic processes (i.e., adaptation).

If variation in levels of investment into parotoid macroglands is indeed driven by natural selection, what selective forces are likely to have been important? The evidence that these glands function to deter predator attacks is conclusive (see above), but the parotoids might have other functions as well. For example, the glands contain hydrophilous glycosaminoglycans that could provide a store of water during long dry periods^30,44^. We doubt the importance of hydroregulation, however, because the two regions where parotoids were largest (Maui wet-side and New South Wales; Fig. 2a) experience moister climates than most of the other regions from which toads were sampled (see^45^ for climatic data). Thus, the most likely selective force driving geographic variation in size of the parotoid glands involves defence against predators.

We first consider the case of males, because their investment into toxins should be driven only by vulnerability to predators of adult toads (whereas investment by females reflects the need for additional allocation to the offspring). The overall pattern for males is straightforward, with low investment in the native range and on the dry sides of Hawai’ian islands, and higher everywhere else (Fig. 2a). Increased parotoid size in Australian populations is consistent with the putatively higher predation pressure in these areas (see above), but available information about predation on cane toads in Hawai’i is too meagre to suggest an explanation for the higher investment into parotoids on wet-side Maui populations.

A female toad’s investment into toxin stores should incorporate two components: the amount needed for her own defence against predation, and the toxins that she needs to allocate to her eggs^28^. However, the optimal allocation of toxins to eggs is complicated by geographic variation in the incidence of cannibalism, which is frequent in Australian cane toads^46,47^ but is infrequent in the native range (DeVore et al., unpublished data). Cannibalistic conspecific tadpoles locate eggs by detecting toxins exuded during late stages of egg development^46^; and thus, a higher investment of toxins into the eggs may render a female’s offspring more vulnerable to cannibalistic attack by conspecifics but less vulnerable to predation by other taxa such as fish and turtles^48,49^.

The disparity between parotoid sizes in female versus male cane toads offers an approximate index of the allocation of toxins to eggs. That disparity is low in the native range (where local predators have evolved to tolerate bufonid toxins, reducing the effectiveness of toxins as a defence) and in Queensland (where larvae are highly cannibalistic, and most clutches are laid in waterbodies that already contain such larvae). However, females from Maui and the dry side of O’ahu also have relatively small parotoids (Fig. 2a), for no obvious reason. In contrast, the sexual disparity in favour of females is maximal in New South Wales, where cannibalism may be infrequent at the invasion front (due to low densities of toads) and embryonic development is slow (due to low water temperatures), increasing the duration of time for which eggs are vulnerable to other types of predators. Field studies are needed to identify selective forces on optimal allocation of toxins to eggs, by quantifying rates of predation (including cannibalism) of toad eggs across the cane toads’ geographic range.

Throughout the manuscript, we have considered cane toad parotoid gland morphology as a proxy for toxin production, and hence anti-predator defence capabilities. While it is likely that toads with larger glands produce more toxin by volume (and invest more energy in doing so), it is possible that divergent selective forces are operating on toxicity or chemical composition of the macrogland secretions as well. For example, in environments where toads frequently encounter naïve predators (i.e., the invaded range), they may require greater volumes of toxin if they exude often as a deterrent in response to harassment. Conversely, in habitats where predators are resistant to toad toxins^20^, as is expected with co-evolved predators in the native-range, selection may act to increase toxicity or alter the composition of parotoid exudate, leading to reciprocal adaptation between predator and prey. Previous work has shown that local adaptation to predation regime or climatic factors can influence both the chemical composition of amphibian toxins, and their potency^50,51^. Therefore, comparisons of the parotoid secretions of cane toads throughout their native and invasive ranges would be an illuminating avenue for future research, and may provide a more nuanced picture of the evolution of anti-predator investments during colonization of new habitats.

In summary, invasive species provide excellent models for studying anti-predator adaptations; the invader arrives with phenotypes that have evolved elsewhere, and may experience different selective forces in colonized habitats than in their native range. The fitness benefits of investment into anti-predator traits can vary greatly in novel environments, generating selection to fine-tune levels of investment depending on cost–benefit trade-offs in local environments. By comparing introduced populations to those within the native range, we can study phenotypic adaptations to geographically variable predation regimes, and explore rapid evolution in response to novel selection pressures. Research on invasive species also allows us to explore the impacts of range spread on phenotypic traits, with investment into anti-predator traits likely trading off with dispersal rate as well as with protection against predators. Overall, strong geographic variation in size, shape and sexual dimorphism of the parotoid macroglands in cane toads supports the hypothesis that investment into defence against predation is fashioned by a complex interplay among selective forces, acting across multiple life-history stages, and that shifts in selective forces can rapidly change investment optima, and thus modify heritable variation in allocation of resources towards anti-predator adaptations.

## Materials and methods

### Study system

Many bufonids (“true toads”) possess paired postorbital macroglands known as parotoids^18,52^ that produce cardiotonic steroids such as bufogenins and bufotoxins^50,53–55^. These toxins have cardio-acceleratory and vasopressor effects on vertebrates^52,56^, such that ingestion can be lethal for predators^57–59^. In cane toads (*Rhinella marina*), this toxin is contained both within the parotoids and a series of smaller glands that are distributed on the dorsal surface and limbs^17^. The cane toad uses the toxin as a passive defence, exuding it when stressed (Fig. 1); predators also may be poisoned by biting into the glands.

The cane toad was introduced from French Guiana to Puerto Rico in the early 1900s, and from there to Hawai’i in 1932, and from there to Australia in 1935 (see^60^ for genetic evidence of this translocation route). Since these introductions, the cane toad has become widely distributed across Hawai’i^25^ and Australia^43,61,62^. Invertebrate predators of eggs and larvae are common in both countries, but predation on adult toads is higher in Australia than in Hawai’i^19,25^.

### Specimen capture and collection sites

From August 2013 to September 2016 we collected adult cane toads (≥ 90 mm snout-vent length, = SVL: see^36,37^) from sites in their native range in French Guiana (N = 302 toads), across their invaded Australian range (QLD, N = 504; NT, N = 929; WA, N = 845; NSW, N = 182) and the Pacific islands of Hawai’i, O’ahu and Maui. We treated the “wet” and “dry” sides of the Hawai'ian islands as separate sampling units, because the climatic divergence between windward and leeward shores within an island is greater than inter-island differences in climatic regimes^25,63^; sample sizes Hawai’i dry-side N = 286, wet-side 244; Maui dry-side N = 95, wet-side 66; O’ahu dry-side N = 126, wet-side 200). Toads were captured by hand, and we used Vernier callipers (± 0.1 mm) to measure the SVL of the toad, and the length (PL) and width (PW) of its right parotoid macrogland. We also measured limb lengths with a plastic ruler (see^36,37^ for details). Sex was determined by secondary sexual characteristics^64^.

### Effect of toxin expulsion on dimensions of the parotoids

If expulsion of toxin alters the size or shape of the parotoids, measurements of these glands on field-caught toads might be affected by local rates of predator attack. Such an effect could introduce significant variation into data for any given population, weakening our ability to detect broader patterns. As part of another study, we manually expressed toxins from the parotoid macroglands of 16 toads, and sham-manipulated another 20 animals to serve as controls. The two groups were matched for overall body sizes. Following toxin removal treatment, toads were radio-tracked for 5 days and then recaptured and their glands and body size measured (see^16^ for details).

### Assessment of heritability of parotoid morphology

Australian toads captured from the wild were brought to our field station 66 km southeast of Darwin, NT (12°37′S 131°18′E) where they were bred. We raised the resultant offspring under standard conditions, to reduce the influence of environmental factors on morphology. The adult toads were collected from three sites in north-eastern Queensland (Townsville, 19°26′S 146°82′E, Innisfail, 17°52′S 146°03′E, and Tully, 17°93′S 145°92′E) and four sites in northern Western Australia (El Questro, 16°00′S 127°98′E, Purnululu, 17°53′S 128°40′E, Wyndham, 15°46′S 128°10′E, and Oombulgurri, 15°18′S 127°84′E). Toads had been present in all of the QLD sites for > 70 years, but had only recently colonized the WA sites (< 2 years^65^). We induced spawning by injection of leuprorelin acetate (Lucrin; Abbott Australasia; using 1 mL of Lucrin diluted 1:20 with saline) and raised the progeny in captivity using the protocols described by^66^. After post-metamorphic toads attained body lengths > 20 mm, we toe-clipped them for identification (a procedure that causes minimal stress to the animals^67^) and kept them in outdoor enclosures in groups of 30 (with mixed parental origins). We raised “common garden” toads from 31 egg clutches (16 QLD, 15 WA) totalling 489 offspring (287 QLD, 202 WA). The offspring were measured three times (at approximately 8, 14, and 17 months of age); 196 animals (132 from QLD, 64 from WA) had reached adult size (> 90 mm SVL) by the end of this study. Our heritability analyses are based on data from 61 parents and 317 offspring > 60 mm SVL, each measured one to three times.

### Calculations and statistical analyses

#### Morphology of the parotoid glands

To obtain an estimate of overall size of the glands, we calculated an approximate area by treating parotoids as rectangular in shape (length*width). That method slightly overestimates area for rounded shapes but the error is small, and is similar among all samples (calculating the area as a circle, based on mean radius, yields a score that is almost perfectly correlated with the rectangular approximation: r^2^ = 0.997). We then ln-transformed values of parotoid area and body length to achieve normality, and regressed ln parotoid area against ln body length to obtain residual scores (deviations from the average parotoid size expected at any given body length) that we used as indices of relative parotoid size (i.e., a high score represents a larger-than-usual parotoid for an animal of that body length). To quantify shape of the parotoids, we divided the width measurement by the length measurement; thus, high scores indicate more rounded shapes. We did not correct for allometry in this measure because our index of parotoid shape was not strongly related to body length in either sex (r^2^ = 0.006 for females, r^2^ < 0.0001 for males).

For analyses of geographic variation, we recognized 11 major regions (although we retained information on collection sites within each region, and used this as a random factor in our statistical analyses to avoid pseudoreplication): (1) all French Guiana sites; (2) Wet (windward) sides of each of three Hawai’ian islands (Hawai’i, O’ahu, Maui); (3) Dry (leeward, rain shadow) sides of each of the same three islands; and four states within Australia: (4) Queensland, (5) New South Wales, (6) Northern Territory, and (7) Western Australia. Table 1 shows sample sizes and associated information for all 54 collection sites. We compared parotoid dimensions between sexes and among regions using a two-factor ANOVA with sex and region as factors, and population as a random variable (to avoid treating toads from the same collection site as statistically independent). All other analyses were performed using JMP 14 software (SAS Institute, Cary, NC). We assessed residuals from all analyses to detect violations of assumptions.

**Table 1:** Sample sizes, body sizes and parotoid sizes of adult cane toads (*Rhinella marina*) collected from the field for the present study.

Sex	Location	Region	Latitude	Longitude	N	Mean SVL ± SE (mm)	Mean Parotoid Length ± SE (mm)	Mean Parotoid Width ± SE (mm)
Fem	Border Ranges	Australia (NSW)	−28.407525	153.08541	33	110.1 ± 2.1	26.7 ± 0.7	19.5 ± 0.5
Fem	Borroloola	Australia (NSW)	−16.6913889	136.2769444	13	104.1 ± 4.0	23.5 ± 1.0	19.0 ± 1.0
Fem	Broom's Head	Australia (NSW)	−29.608228	153.3358185	19	110.6 ± 2.7	27.1 ± 0.9	21.8 ± 0.9
Fem	Cairns	Australia (QLD)	−16.920334	145.7708595	41	108.2 ± 1.1	25.8 ± 0.4	19.2 ± 0.4
Fem	Caroline Pool	Australia (WA)	−18.226814	127.759649	38	125.3 ± 1.3	30.6 ± 0.6	23.3 ± 0.4
Fem	Charters Towers	Australia (QLD)	−20.050480	146.2538153	12	110.6 ± 2.6	26.4 ± 1.1	20.0 ± 0.8
Fem	Darwin Botanic Gardens	Australia (NT)	−12.444302	130.836995	18	102.3 ± 2.9	24.0 ± 1.1	18.0 ± 0.8
Fem	Durack River Crossing	Australia (WA)	−15.94548429	127.2213364	38	115.6 ± 1.6	27.6 ± 0.6	22.5 ± 0.5
Fem	El Questro Station	Australia (WA)	−16.008438	127.979811	65	113.3 ± 1.4	27.1 ± 0.5	20.0 ± 0.3
Fem	Ellenbrae Station	Australia (WA)	−15.9743433	127.0621375	26	121.3 ± 2.0	28.9 ± 0.6	23.4 ± 0.5
Fem	Fogg Dam	Australia (NT)	−12.568032	131.309507	91	123.3 ± 1.6	29.4 ± 0.5	23.4 ± 0.4
Fem	Innisfail	Australia (QLD)	−17.524681	146.0323287	54	109.2 ± 1.4	25.2 ± 0.5	19.1 ± 0.4
Fem	Kakadu	Australia (NT)	−13.092293	132.393766	72	120.6 ± 1.2	27.7 ± 0.4	22.5 ± 0.3
Fem	Katherine	Australia (NT)	−14.464967	132.2642561	38	114.9 ± 2.8	27.5 ± 0.8	21.2 ± 0.7
Fem	Kununurra	Australia (WA)	−15.773546	128.739196	20	113.8 ± 2.6	26.8 ± 0.7	20.8 ± 0.6
Fem	Kyogle	Australia (NSW)	−28.620356	153.0040612	22	117.3 ± 3.1	29.5 ± 1.2	22.2 ± 0.8
Fem	Leaning Tree Lagoon	Australia (NT)	−12.709088	131.420238	109	115.7 ± 1.7	27.2 ± 0.5	20.9 ± 0.5
Fem	Litchfield	Australia (NT)	−13.293538	130.846388	39	106.0 ± 2.3	24.9 ± 0.7	20.0 ± 0.5
Fem	Mount Isa	Australia (QLD)	−20.7247053	139.4974616	29	122.9 ± 2.5	30.5 ± 0.9	23.5 ± 0.8
Fem	Oombulgurri	Australia (WA)	−15.180417	127.8450389	102	120.2 ± 1.1	28.0 ± 0.3	21.8 ± 0.3
Fem	Palm Hand Creek	Australia (WA)	−18.026354	127.804047	25	118.5 ± 1.7	28.7 ± 0.5	21.9 ± 0.5
Fem	Pine Creek	Australia (NT)	−13.8248434	131.8349128	12	114.5 ± 3.3	27.3 ± 1.0	20.0 ± 0.8
Fem	Purnululu	Australia (WA)	−17.529752	128.400838	59	107.0 ± 1.6	24.9 ± 0.4	18.7 ± 0.4
Fem	Timber Creek	Australia (NT)	−15.6502331	130.4777132	40	112.4 ± 1.6	26.2 ± 0.5	21.3 ± 0.4
Fem	Townsville	Australia (QLD)	−19.257627	146.8178707	85	107.3 ± 0.9	24.6 ± 0.3	19.2 ± 0.2
Fem	Tully	Australia (QLD)	−17.932869	145.9235556	48	105.8 ± 1.8	25.1 ± 0.6	18.7 ± 0.5
Fem	Wave Hill	Australia (NT)	−17.7158333	130.9457445	17	96.6 ± 1.2	21.9 ± 0.6	17.1 ± 0.4
Fem	Wyndham	Australia (WA)	−15.464803	128.1001426	59	114.3 ± 1.9	26.6 ± 0.5	20.6 ± 0.5
Fem	Yamba	Australia (NSW)	−29.437827	153.3602722	23	107.1 ± 2.6	26.0 ± 0.9	20.1 ± 0.5
Male	Border Ranges	Australia (NSW)	−28.407525	153.08541	35	101.7 ± 1.0	23.6 ± 0.4	16.6 ± 0.3
Male	Borroloola	Australia (NSW)	−16.6913889	136.2769444	29	105.5 ± 1.4	25.1 ± 0.5	18.4 ± 0.3
Male	Broom's Head	Australia (NSW)	−29.608228	153.3358185	17	104.5 ± 1.5	26.2 ± 0.8	18.3 ± 0.5
Male	Cairns	Australia (QLD)	−16.920334	145.7708595	38	102.4 ± 1.0	24.6 ± 0.4	17.3 ± 0.3
Male	Caroline Pool	Australia (WA)	−18.226814	127.759649	55	115.5 ± 0.8	28.8 ± 0.4	20.6 ± 0.3
Male	Charters Towers	Australia (QLD)	−20.050480	146.2538153	15	104.4 ± 1.7	26.3 ± 0.7	17.1 ± 0.4
Male	Darwin Botanic Gardens	Australia (NT)	−12.444302	130.836995	22	107.1 ± 2.0	25.2 ± 0.9	18.2 ± 0.4
Male	Durack River Crossing	Australia (WA)	−15.94548429	127.2213364	43	110.0 ± 0.9	26.7 ± 0.4	19.8 ± 0.3
Male	El Questro Station	Australia (WA)	−16.008438	127.979811	41	106.7 ± 1.4	25.4 ± 0.5	17.9 ± 0.3
Male	Ellenbrae Station	Australia (WA)	−15.9743433	127.0621375	24	114.4 ± 1.6	27.7 ± 0.5	19.9 ± 0.3
Male	Fogg Dam	Australia (NT)	−12.568032	131.309507	65	116.0 ± 1.4	28.0 ± 0.4	20.2 ± 0.3
Male	Innisfail	Australia (QLD)	−17.524681	146.0323287	34	105.1 ± 1.3	26.3 ± 0.6	17.4 ± 0.3
Male	Kakadu	Australia (NT)	−13.092293	132.393766	74	116.9 ± 0.9	27.8 ± 0.3	20.0 ± 0.2
Male	Katherine	Australia (NT)	−14.464967	132.2642561	34	111.5 ± 2.0	26.4 ± 0.5	19.9 ± 0.4
Male	Kununurra	Australia (WA)	−15.773546	128.739196	26	107.3 ± 1.5	24.7 ± 0.6	18.5 ± 0.4
Male	Kyogle	Australia (NSW)	−28.620356	153.0040612	17	103.4 ± 1.3	24.9 ± 0.5	17.0 ± 0.4
Male	Leaning Tree Lagoon	Australia (NT)	−12.709088	131.420238	173	110.8 ± 0.6	27.0 ± 0.2	19.4 ± 0.2
Male	Litchfield	Australia (NT)	−13.293538	130.846388	33	109.2 ± 1.9	25.8 ± 0.5	19.9 ± 0.4
Male	Mount Isa	Australia (QLD)	−20.7247053	139.4974616	37	107.1 ± 1.5	27.5 ± 0.6	18.6 ± 0.5
Male	Oombulgurri	Australia (WA)	−15.180417	127.8450389	85	110.1 ± 0.9	26.8 ± 0.3	19.3 ± 0.2
Male	Palm Hand Creek	Australia (WA)	−18.026354	127.804047	14	109.7 ± 3.1	26.0 ± 1.2	19.4 ± 0.6
Male	Pine Creek	Australia (NT)	−13.8248434	131.8349128	7	105.5 ± 3.5	26.0 ± 1.4	17.8 ± 0.6
Male	Purnululu	Australia (WA)	−17.529752	128.400838	41	104.1 ± 1.2	24.7 ± 0.4	17.2 ± 0.3
Male	Timber Creek	Australia (NT)	−15.6502331	130.4777132	22	109.5 ± 1.2	25.4 ± 0.5	19.6 ± 0.3
Male	Townsville	Australia (QLD)	−19.257627	146.8178707	68	101.4 ± 0.8	24.3 ± 0.3	17.0 ± 0.2
Male	Tully	Australia (QLD)	−17.932869	145.9235556	43	98.8 ± 1.1	23.5 ± 0.4	16.5 ± 0.2
Male	Wave Hill	Australia (NT)	−17.7158333	130.9457445	57	107.3 ± 1.1	25.7 ± 0.4	18.9 ± 0.2
Male	Wyndham	Australia (WA)	−15.464803	128.1001426	47	104.8 ± 1.1	24.7 ± 0.4	17.4 ± 0.3
Male	Yamba	Australia (NSW)	−29.437827	153.3602722	17	95.7 ± 1.4	23.1 ± 0.6	15.9 ± 0.5
Fem	Kourou Atlantis	French Guiana	5.15715438	−52.64640525	12	105.5 ± 2.4	23.5 ± 0.8	18.7 ± 0.7
Fem	Remire-Montjoly Beach N	French Guiana	4.917072768	−52.26695448	9	120.1 ± 4.1	26.8 ± 0.9	22.0 ± 0.8
Fem	Remire-Montjoly Beach S	French Guiana	4.890092298	−52.25395113	49	110.0 ± 2.1	24.2 ± 0.7	19.7 ± 0.5
Fem	St. Georges	French Guiana	3.900456271	−51.80153087	6	110.6 ± 5.8	25.6 ± 2.1	21.9 ± 1.7
Fem	Yalimapo Beach	French Guiana	5.746320083	−53.94172579	7	135.4 ± 7.5	30.0 ± 1.9	25.9 ± 1.9
Male	Kourou Atlantis	French Guiana	5.15715438	−52.64640525	48	103.9 ± 1.9	23.3 ± 0.7	17.5 ± 0.6
Male	Remire-Montjoly Beach N	French Guiana	4.917072768	−52.26695448	36	106.7 ± 1.3	24.0 ± 0.5	18.0 ± 0.3
Male	Remire-Montjoly Beach S	French Guiana	4.890092298	−52.25395113	88	109.1 ± 1.2	24.6 ± 0.4	18.2 ± 0.3
Male	St. Georges	French Guiana	3.900456271	−51.80153087	40	125.9 ± 3.2	30.2 ± 1.1	23.5 ± 0.7
Male	Yalimapo Beach	French Guiana	5.746320083	−53.94172579	7	126.9 ± 4.1	30.1 ± 1.5	23.0 ± 0.9
Fem	Hilo	Hawai’i (Wet)	19.700250	−155.081687	89	109.3 ± 1.7	25.7 ± 0.6	19.8 ± 0.4
Fem	Pahoa	Hawai’i (Wet)	19.4975033	−154.9508104	13	99.6 ± 2.1	24.8 ± 0.8	18.8 ± 0.6
Fem	Panewa Zoo	Hawai’i (Wet)	19.653754	−155.073764	11	99.6 ± 2.5	22.2 ± 0.6	17.9 ± 0.7
Fem	Tom’s Volcano Ranch	Hawai’i (Wet)	19.5437679	−155.131557	5	101.5 ± 3.7	24.1 ± 1.1	19.4 ± 1.0
Fem	Big Island Country Club	Hawai’i (Dry)	19.822272	−155.844169	9	108.5 ± 2.8	26.8 ± 1.1	20.3 ± 0.6
Fem	King’s Shops	Hawai’i (Dry)	19.916323	−155.88209	48	110.8 ± 1.9	26.3 ± 0.7	19.7 ± 0.5
Fem	Kona	Hawai’i (Dry)	19.5711075	−155.957051	14	125.2 ± 4.6	27.7 ± 1.0	21.2 ± 0.9
Fem	Mauna Kea Golf Course	Hawai’i (Dry)	20.003944	−155.820113	72	121.1 ± 1.4	27.7 ± 0.5	20.6 ± 0.4
Fem	Iao	Maui (Wet)	20.91	−156.49	31	113.0 ± 2.6	28.3 ± 0.8	20.5 ± 0.6
Fem	Wailua	Maui (Wet)	20.8245438	−156.0909414	5	99.3 ± 3.7	24.7 ± 1.3	18.3 ± 1.6
Fem	Kaanapali Golf Course	Maui (Dry)	20.9159	−156.692446	30	112.2 ± 1.6	27.5 ± 0.5	19.6 ± 0.5
Fem	Makena	Maui (Dry)	20.65884	−156.437698	18	115.1 ± 3.6	28.7 ± 1.1	19.4 ± 0.8
Fem	BYU Campus La’ie	O’ahu (Wet)	21.641842	−157.925354	22	117.4 ± 3.2	30.0 ± 0.9	21.5 ± 0.8
Fem	Haiku Gardens	O’ahu (Wet)	21.4167622	−157.8138132	34	108.8 ± 2.0	26.9 ± 0.7	20.2 ± 0.6
Fem	Kailua	O’ahu (Wet)	21.372767	−157.7325974	26	114.1 ± 3.3	26.7 ± 1.0	19.5 ± 0.6
Fem	Kalaeoio Beach Park	O’ahu (Wet)	21.547254	−157.847012	6	130.5 ± 3.4	30.2 ± 1.4	25.4 ± 2.4
Fem	Lyon Arboretum	O’ahu (Wet)	21.332976	−157.801573	4	124.7 ± 6.9	35.6 ± 2.2	23.2 ± 1.6
Fem	Kapolei Regional Park	O’ahu (Dry)	21.335617	−158.077933	35	111.2 ± 1.7	27.9 ± 0.5	20.2 ± 0.5
Fem	Mililani	O’ahu (Dry)	21.46433186	−158.0219578	25	123.3 ± 2.6	31.7 ± 0.9	24.2 ± 0.8
Fem	Wahiawa	O’ahu (Dry)	21.5010495	−158.029227	7	105.3 ± 3.6	23.8 ± 1.3	17.0 ± 0.9
Male	Hilo	Hawai’i (Wet)	19.700250	−155.081687	65	98.0 ± 1.0	22.7 ± 0.3	16.3 ± 0.3
Male	Pahoa	Hawai’i (Wet)	19.4975033	−154.9508104	14	101.8 ± 2.0	25.7 ± 0.9	17.6 ± 0.4
Male	Panaewa Zoo	Hawai’i (Wet)	19.653754	−155.073764	33	103.4 ± 1.2	25.1 ± 0.4	16.9 ± 0.3
Male	Tom’s Volcano Ranch	Hawai’i (Wet)	19.5437679	−155.131557	14	95.1 ± 1.4	22.1 ± 0.5	15.8 ± 0.4
Male	Big Island Country Club	Hawai’i (Dry)	19.822272	−155.844169	30	106.0 ± 1.1	24.8 ± 0.4	16.3 ± 0.3
Male	King’s Shops	Hawai’i (Dry)	19.916323	−155.88209	30	99.5 ± 1.3	22.6 ± 0.5	15.3 ± 0.3
Male	Kona	Hawai’i (Dry)	19.5711075	−155.957051	21	108.5 ± 2.8	24.4 ± 0.9	17.3 ± 0.6
Male	Mauna Kea Golf Course	Hawai’i (Dry)	20.003944	−155.820113	62	110.2 ± 0.9	26.1 ± 0.3	16.5 ± 0.2
Male	Iao	Maui (Wet)	20.91	−156.49	26	113.0 ± 2.6	28.3 ± 0.8	20.5 ± 0.6
Male	Wailua	Maui (Wet)	20.8245438	−156.0909414	4	99.3 ± 3.7	24.7 ± 1.3	18.3 ± 1.6
Male	Kaanapali Golf Course	Maui (Dry)	20.9159	−156.692446	29	108.2 ± 1.8	27.6 ± 0.7	18.4 ± 0.4
Male	Makena	Maui (Dry)	20.65884	−156.437698	18	104.2 ± 1.9	25.7 ± 0.6	16.8 ± 0.4
Male	BYU Campus La’ie	O’ahu (Wet)	21.641842	−157.925354	32	107.8 ± 1.7	26.7 ± 0.6	17.3 ± 0.4
Male	Haiku Gardens	O’ahu (Wet)	21.4167622	−157.8138132	50	105.1 ± 1.0	26.4 ± 0.4	17.7 ± 0.2
Male	Kailua	O’ahu (Wet)	21.372767	−157.7325974	19	107.6 ± 2.2	26.1 ± 0.8	16.5 ± 0.6
Male	Kalaeoio Beach Park	O’ahu (Wet)	21.547254	−157.847012	4	116.2 ± 2.9	32.1 ± 2.0	19.8 ± 0.5
Male	Lyon Arboretum	O’ahu (Wet)	21.332976	−157.801573	3	116.0 ± 2.5	32.0 ± 1.6	21.7 ± 1.0
Male	Kapolei Regional Park	O’ahu (Dry)	21.335617	−158.077933	27	109.7 ± 1.9	29.6 ± 0.9	19.3 ± 0.5
Male	Mililani	O’ahu (Dry)	21.46433186	−158.0219578	24	115.1 ± 0.9	30.0 ± 0.7	20.3 ± 0.3
Male	Wahiawa	O’ahu (Dry)	21.5010495	−158.029227	8	103.3 ± 2.1	24.7 ± 0.7	16.7 ± 0.6

*Fem* female, *SVL* snout-vent length, *NSW* New South Wales, *QLD* Queensland, *WA* Western Australia, *NT* Northern Territory.

#### Common-garden offspring

We measured the animals in the same way as for field-collected specimens, and calculated the same variables as above to describe parotoid size and shape. We excluded measurements of individuals < 60 mm SVL (because of imprecision in measuring such small animals). To estimate heritability and repeatability of size and shape of the parotoids, we used ASREML software (VSN International Ltd., Hemel Hempstead, UK) to run an animal model^68^ incorporating individual ID and family ID as random effects.

### Ethics statement

All procedures in the current study were approved by the University of Sydney Animal Care and Ethics Committee (permit numbers 2013/6075, 2017/1195, and 2018/1441), and carried out in accordance with ARRIVE guidelines and with all other relevant guidelines and regulations. Field work in Hawai’i was conducted with approval from the State of Hawai’i Department of Land and Natural Resources, Division of Forestry and Wildlife (permit numbers Ex15-15 and Ex15-16). This article does not contain any studies performed with human participants performed by any of the authors.

## Data Availability

Data can be found in the Dryad Data Repository at 10.5061/dryad.rn8pk0p8j.

## References

[1] Caro, T. M. *Antipredator Defenses in Birds and Mammals* (University of Chicago Press, 2005).

[2] Emlen DJ (2008). The evolution of animal weapons. Annu. Rev. Ecol. Evol. Syst..

[3] Toledo LF, Sazima I, Haddad CF (2011). Behavioural defences of anurans: An overview. Ethol. Ecol. Evol..

[4] Lima SL, Dill LM (1990). Behavioral decisions made under the risk of predation: A review and prospectus. Can. J. Zool..

[5] Pettorelli N, Coulson T, Durant SM, Gaillard J (2011). Predation, individual variability and vertebrate population dynamics. Oecologia.

[6] Stankowich T (2011). Armed and dangerous: predicting the presence and function of defensive weaponry in mammals. Adapt. Behav..

[7] Longson CG, Joss JMP (2006). Optimal toxicity in animals: Predicting the optimal level of chemical defences. Funct. Ecol..

[8] Relyea RA (2003). Predators come and predators go: The reversibility of predator-induced traits. Ecology.

[9] Tollrian, R. & Harvell, D. *The Ecology and Evolution of Inducible Defenses* (Princeton University Press, 1999).

[10] Daly D, Higginson AD, Chen D, Ruxton GD, Speed MP (2012). Density-dependent investment in costly anti-predator defenses: An explanation for the weak survival benefit of group living. Ecol. Lett..

[11] Kosmala G, Brown GP, Shine R (2020). Thin-skinned invaders: Geographic variation in the structure of the skin among populations of cane toads (*Rhinella marina*). Biol. J. Linn. Soc..

[12] Duellman, W. E. & Trueb, L. *Biology of Amphibians* (McGraw-Hill, 1994).

[13] Wells, K. *The Ecology and Behavior of Amphibians* (University of Chicago Press, 2007).

[14] König E, Bininda-Emonds ORP, Shaw C (2014). The diversity and evolution of anuran skin peptides. Peptides.

[15] Hettyey A, Tóth Z, Van Buskirk J (2014). Inducible chemical defences in animals. Oikos.

[16] Blennerhasset R, Bell-Anderson K, Shine R, Brown GP (2019). The cost of chemical defence: The impact of toxin depletion on growth and behaviour of cane toads (*Rhinella marina*). Proc. R. Soc. B..

[17] Chen W, Hudson CM, DeVore JL, Shine R (2017). Sex and weaponry: The distribution of toxin-storage glands on the bodies of male and female cane toads (*Rhinella marina*). Ecol. Evol..

[18] O’Donohoe MA (2019). Diversity and evolution of the parotoid macrogland in true toads (Anura: Bufonidae). Zool. J. Linn. Soc..

[19] Shine R (2010). The ecological impact of invasive cane toads (*Bufo marinus*) in Australia. Q. Rev. Biol..

[20] Ujvari B (2013). Isolation breeds naivety: island living robs Australian varanid lizards of toad-toxin immunity via four-base-pair mutation. Evolution.

[21] Pearcy A (2011). Selective feeding in Keelback snakes *Tropidonophis mairii* in an Australian wetland. Aust. Zool..

[22] Llewelyn J (2018). Behavioural responses of an Australian colubrid snake (*Dendrelaphis punctulatus*) to a novel toxic prey item (the Cane Toad *Rhinella marina*). Biol. Invasions.

[23] van Bocxlaer I (2010). Gradual adaptation toward a range-expansion phenotype initiated the global radiation of toads. Science.

[24] Hudson CM, Vidal-García M, Murray TG, Shine R (2020). The accelerating anuran: evolution of locomotor performance in cane toads (*Rhinella marina*, Bufonidae) at an invasion front. Proc. R. Soc. B.

[25] Ward-Fear G, Greenlees MJ, Shine R (2016). Toads on lava: spatial ecology and habitat use of invasive cane toads (*Rhinella marina*) in Hawai’i. PLoS ONE.

[26] Ward-Fear G, Pearson DJ, Brown GP, Shine R (2016). Ecological immunization: *in situ* training of free-ranging predatory lizards reduces their vulnerability to invasive toxic prey. Biol. Lett..

[27] Crossland MR, Brown GP, Anstis M, Shilton C, Shine R (2008). Mass mortality of native anuran tadpoles in tropical Australia due to the invasive cane toad (*Bufo marinus*). Biol. Conserv..

[28] Hayes RA, Crossland MR, Hagman M, Capon RJ, Shine R (2009). Ontogenetic variation in the chemical defences of cane toads (*Bufo marinus*): Toxin profiles and effects on predators. J. Chem. Ecol..

[29] Hagman M, Hayes RA, Capon RJ, Shine R (2009). Alarm cues experienced by cane toad tadpoles affect post-metamorphic morphology and chemical defences. Funct. Ecol..

[30] Üveges B (2017). Age-and environment-dependent changes in chemical defences of larval and post-metamorphic toads. BMC Evol. Biol..

[31] Üveges B (2019). Chemical defense of toad tadpoles under risk by four predator species. Ecol. Evol..

[32] Bókony V, Üveges B, Verebélyi V, Ujhegyi N, Móricz ÁM (2019). Toads phenotypically adjust their chemical defences to anthropogenic habitat change. Sci. Rep..

[33] Hettyey A (2019). Predator-induced changes in the chemical defence of a vertebrate. J. Anim. Ecol..

[34] Hudson, C. M, Brown, G. P., Stuart, K. & Shine, R. Sexual and geographic divergence in head widths of invasive cane toads, *Rhinella marina* (Anura: Bufonidae) is driven by both rapid evolution and plasticity. *Biol*. *J*. *Linn*. *Soc*. **124,** 188–199 (2018).

[35] Phillips BL, Brown GP, Webb JK, Shine R (2006). Invasion and the evolution of speed in toads. Nature.

[36] Hudson CM, McCurry MR, Lundgren P, McHenry CR, Shine R (2016). Constructing an invasion machine: The rapid evolution of a dispersal-enhancing phenotype during the cane toad invasion of Australia. PLoS ONE.

[37] Hudson CM, Brown GP, Shine R (2016). It is lonely at the front: Contrasting evolutionary trajectories in male and female invaders. R. Soc. Open Sci..

[38] Brown G, Kelehear C, Shine R (2013). The early toad gets the worm: Cane toads at an invasion front benefit from higher prey availability. J. Anim. Ecol..

[39] Shine R, Brown GP, Phillips BL (2011). An evolutionary process that assembles phenotypes through space rather than time. Proc. Natl Acad. Sci. USA.

[40] Phillips B, Shine R (2005). The morphology, and hence impact, of an invasive species (the cane toad, *Bufo marinus*) changes with time since colonization. Anim. Conserv..

[41] Roff DA (2008). Comparing sire and dam estimates of heritability: Jackknife and likelihood approaches. Heredity.

[42] Kliber A, Eckert CG (2005). Interaction between founder effect and selection during biological invasion in an aquatic plant. Evolution.

[43] Shine, R. *Cane Toad Wars* (University of California Press, 2018).

[44] Toledo RC, Jared C (1993). Cutaneous adaptations to water balance in amphibians. Comp. Biochem. Physiol. A.

[45] Kosmala G, Brown GP, Shine R, Christian K (2020). Skin resistance to water gain and loss has changed in cane toads (*Rhinella marina*) during their Australian invasion. Ecol. Evol..

[46] Crossland MR, Shine R (2011). Cues for cannibalism: Cane toad tadpoles use chemical signals to locate and consume conspecific eggs. Oikos.

[47] DeVore JL, Crossland M, Shine R (2020). Tradeoffs affect the adaptive value of plasticity: Stronger cannibal-induced defenses incur greater costs in toad larvae. Ecol. Monogr..

[48] Greenlees MJ, Shine R (2011). Impacts of eggs and tadpoles of the invasive cane toad (*Bufo marinus*) on aquatic predators in tropical Australia. Austral Ecol..

[49] Somaweera R, Crossland MR, Shine R (2011). Assessing the potential impact of invasive cane toads on a commercial freshwater fishery in tropical Australia. Wildl. Res..

[50] Cao Y, Cui K, Pan H, Wu J, Wang L (2019). The impact of multiple climatic and geographic factors on the chemical defences of Asian toads (*Bufo gargarizans* Cantor). Sci. Rep..

[51] Hague, M. T. J., Stokes, A. N., Feldman, C. R., Brodie, E. D. Jr. & Brodie, E. D. III. The geographic mosaic of arms race coevolution is closely matched to prey population structure. *Evol*. *Lett*. **4,** 317–332 (2020). 10.1002/evl3.184 PMC7403720 32774881

[52] Jared C (2009). Parotoid macroglands in toad (*Rhinella jimi*): Their structure and functioning in passive defence. Toxicon.

[53] Toledo RC, Jared C (1995). Cutaneous granular glands and amphibian venoms. Comp. Biochem. Physiol. A.

[54] Maciel NM (2003). Composition of indolealkylamines of *Bufo rubescens* cutaneous secretions compared to six other Brazilian bufonids with phylogenetic implications. Comp. Biochem. Physiol. B.

[55] Sciani JM, Angeli CB, Antoniazzi MM, Jared C, Pimenta DC (2013). Differences and similarities among parotoid macrogland secretions in South American toads: A preliminary biochemical delineation. Sci. World J..

[56] Habermehl, G. *Venomous Animals and Their Toxins* (Springer-Verlag, 1981).

[57] Garrett CM, Boyer DM (1993). *Bufo marinus* (cane toad) predation. Herpetol. Rev..

[58] Pineau X, Romanoff C (1995). Envenomation of domestic carnivores. Rec. Méd. Vét..

[59] Sakate, M. & Lucas de Oliveira, P. C. Toad envenoming in dogs: effects and treatment. *J. Venom. Anim. Toxins* **6,** 52–62 (2000).

[60] Slade RW, Moritz C (1998). Phylogeography of *Bufo marinus* from its natural and introduced ranges. Proc. R. Soc. B.

[61] Urban MC, Phillips BL, Skelly DK, Shine R (2007). The cane toad’s (*Chaunus* [*Bufo*] *marinus*) increasing ability to invade Australia is revealed by a dynamically updated range model. Proc. R. Soc. B.

[62] Urban M, Phillips BL, Skelly DK, Shine R (2008). A toad more traveled: The heterogeneous invasion dynamics of cane toads in Australia. Am. Nat..

[63] Nullet D, Juvik JO, Wall A (1995). A Hawaiian mountain climate cross-section. Clim. Res..

[64] Kelehear C, Shine R (2019). Non-reproductive male cane toads (*Rhinella marina*) withhold sex-identifying information from their rivals. Biol. Lett..

[65] Shine R, Everitt C, Woods D, Pearson DJ (2018). An evaluation of methods used to cull invasive cane toads in tropical Australia. J. Pest Sci..

[66] Phillips BL, Kelehear C, Pizzatto L, Brown GP, Barton D, Shine R (2010). Parasites and pathogens lag behind their host during periods of host range-advance. Ecology.

[67] Hudson CM, Brown GP, Shine R (2017). Effects of toe-clipping on growth, body condition, and locomotion of cane toads (*Rhinella marina*). Copeia.

[68] Wilson AJ (2010). An ecologist’s guide to the animal model. J. Anim. Ecol..

